# Curcumin Suppresses Gelatinase B Mediated Norepinephrine Induced Stress in H9c2 Cardiomyocytes

**DOI:** 10.1371/journal.pone.0076519

**Published:** 2013-10-07

**Authors:** Shrey Kohli, Aastha Chhabra, Astha Jaiswal, Yashika Rustagi, Manish Sharma, Vibha Rani

**Affiliations:** 1 Department of Biotechnology, Jaypee Institute of Information Technology, Noida, Uttar Pradesh, India; 2 Peptide and Proteomics Division, Defence Institute of Physiology and Allied Sciences (DIPAS), DRDO, Delhi, India; National Center for Scientific Research Demokritos, Greece

## Abstract

**Background:**

Extracellular matrix (ECM) remodeling facilitates biomechanical signals in response to abnormal physiological conditions. This process is witnessed as one of the major effects of the stress imposed by catecholamines, such as epinephrine and norepinephrine (NE), on cardiac muscle cells. Matrix metalloproteinases (MMPs) are the key proteases involved in degradation of the ECM in heart.

**Objectives:**

The present study focuses on studying the effect of curcumin on Gelatinase B (MMP-9), an ECM remodeling regulatory enzyme, in NE-induced cardiac stress. Curcumin, a bioactive polyphenol found in the spice turmeric, has been studied for its multi-fold beneficial properties. This study focuses on investigating the role of curcumin as a cardio-protectant.

**Methods:**

H9c2 cardiomyocytes were subjected to NE and curcumin treatments to study the response in stress conditions. Effect on total collagen content was studied using Picrosirus red staining. Gelatinase B activity was assessed through Gel-Diffusion Assay and Zymographic techniques. RT-PCR, Western Blotting and Immunocytochemistry were performed to study effect on expression of gelatinase B. Further, the effect of curcumin on the localization of NF-κB, known to regulate gelatinase B, was also examined.

**Results:**

Curcumin suppressed the increase in the total collagen content under hypertrophic stress and was found to inhibit the in-gel and *in-situ* gelatinolytic activity of gelatinase B. Moreover, it was found to suppress the mRNA and protein expression of gelatinase B.

**Conclusions:**

The study provides an evidence for an overall inhibitory effect of curcumin on Gelatinase B in NE-induced hypertrophic stress in H9c2 cardiomyocytes which may contribute in the prevention of ECM remodeling.

## Introduction

The catecholamines, Epinephrine and Norepinephrine (NE), have been demonstrated to pose stress conditions on the cardiac cells deteriorating their structure and function [1]. NE, an adrenergic agonist has been shown to induce stress on the heart with and without adrenoreceptor blockade even in micromolar concentrations [2]. Severe toxic insults cause cell death instantly but in an early response to mild stimuli, hypertrophy occurs and involves enlargement of cardiomyocytes as well as activation of counter-regulatory mechanisms including overexpression of fetal genes such as Atrial Natriuretic factor (ANF). Prolonged hypertrophy leads to cardiotoxicity, causing cell death and ultimately cardiac failure [3]. One major effect of such stress is remodeling of the myocardial extracellular matrix (ECM) known to facilitate the biomechanical signals in response to abnormal physiological conditions [4]. ECM turnover is regulated by matrix metalloproteinases (MMPs) which are a family of calcium dependent, zinc containing, substrate specific endopeptidases, subdivided into six major classes including collagenases, gelatinases, stromelysins, matrilysins, membrane type MMPs and other unclassified MMPs [5,6]. The primary function of these proteases is to degrade ECM proteins. A comparative overview of the functional properties of the 23 members reported in humans till date has been described by our group previously [7]. ECM remodeling involves degradation as well as increased synthesis of collagen. Collagen turnover is found to be upregulated due to an imbalance between the rate of its degradation and synthesis under stress leading to deviations from its normal content. The rate of collagen synthesis overrides the rate of its degradation. In response to this, an upregulated enzymatic activity of MMPs mainly MMP-2 and 9 (Gelatinase A & B respectively) is observed [8,9]. Although gelatinase A has been reported to play a role in cardiac disease, we have focused our study to gelatinase B owing to its meager mechanistic information. Further, identifying a prospective therapeutic strategy for its inhibition would be of great benefit. *Curcuma longa*, a traditional Indian medicinal herb, has been widely applied in clinical therapy for centuries. Curcumin, the principal curcuminoid obtained from this herb, is found to have multifold pharmaceutical properties including anti-microbial, anti-inflammatory, anti-ageing, anti-proliferative, anti-oxidative, neuroprotective and cardioprotective [10-17]. Recently, it has been established that curcumin attenuates maladaptive cardiac repair and improves cardiac function by reducing degradation of ECM [18]. Our earlier studies have evaluated its cardioprotective potential by targeting the transcriptional pathway regulating the re-expression of fetal cardiac gene program [19]. We further explore our research to analyze the effect of curcumin on the proteins involved in the ECM remodeling in NE-induced stress in cardiomyocytes. To our knowledge, the effect of curcumin on gelatinases in this condition has not been addressed till date. This study examines the influence of exogenous curcumin on gelatinase B in NE-induced stress using H9c2 cardiomyocytes as the model system [20]. Our studies reveal for the first time that curcumin suppresses the upregulated activity and expression of gelatinase B due to NE-induced stress in H9c2 cardiomyocytes.

## Methods

### Cell culture and Curcumin Treatment

Embryonic Rat Heart-derived H9c2 cells (NCCS, Pune, India) were cultured and hypertrophic stress was induced using 2 µM NE as previously described [19]. A concentration of 8 µM curcumin as reported earlier by our group was added simultaneous to the NE induction [19]. Cell size was studied and then analyzed by NIH ImageJ. Additionally, forward scatter of the cells was recorded in flow cytometry experiments using FACS Calibur (BD Biosciences, USA).

### RT-PCR

Total RNA was extracted using TRIzol reagent (Ambion) after treatment under different experimental conditions and cDNA was synthesized using oligo-dT primers. The product was PCR amplified under semi-quantitative conditions using gene-specific primers (Table 1). To validate the results, qRT-PCR was performed in triplicates using SYBR Green chemistry and fold change in Gene expression was calculated using ΔΔCt Method after normalizing the data to β-Actin reference gene.

**Table 1:** Primers for RT-PCR.

**Gene**	**Sequence**	**T_a_**
ANF	(F) 5'-CTGCTAGACCACCTGGAGGA-3'	60°C
	(R) 5'-AAGCTGTTGCAGCCTAGTCC-3'	
MMP-9	(F) 5’-CACCGCTCACCTTCACCCG-3’	66°C
	(R) 5’-TGCCGAGTTGCCCCCAGTTA-3’	
β-Actin	(F) 5'-CATCGTACTCCTGCTTGCTG-3'	57.5°C
	(R) 5'-CCTCTATGCCAACACAGTGC-3'	

### Estimation of collagen content

H9c2 cells were treated with NE and curcumin. Followed by PBS wash and methanol fixation, cells were stained with 0.1% Sirius Red F3BA in saturated picric acid (w/v) for 1 hr at room temperature. The collagen bound stain was further eluted with 0.1 N NaOH for 5 min. The absorbance of eluted stain was recorded at 540 nm in a microplate reader (Bio-Rad Labs). A standard curve was plotted as quantity of collagen versus absorbance and the collagen content of samples was estimated.

### Extraction of total cell protein

Cell pellet was washed with ice-cold PBS and lysed using RIPA buffer (20 mM Tris-HCl (pH 7.5), 150 mM NaCl, 1 mM Na _2_EDTA, 1 mM EGTA, 1% NP-40, 0.25% sodium deoxycholate, 2.5 mM sodium pyrophosphate, 1 mM Na _3_VO_4_, Protease Inhibitors Cocktail) in ice for 1 hr for the extraction of total cell protein. The total protein was obtained after centrifugation at 13000g for 15 min in a refrigerated centrifuge. Quantitation of total protein obtained was done using Bicinchoninic acid (BCA) assay.

### Extraction of Nuclear and Cytosolic protein

Nuclear and cytosolic protein extracts of the cells were prepared by incubating the cells firstly with Buffer A (20mM HEPES, 20% Glycerol, 10mM NaCl, 1.5 mM MgCl_2_, 0.2mM EDTA, 0.1% Triton X-100, 1mM DTT, 100mM PMSF, Protease Inhibitors Cocktail) in ice for 15 minutes. This was followed by centrifugation at low speed. Cytosolic extract was obtained in the supernatant and the pellet obtained was resuspended in ice-chilled Buffer B (20mM HEPES, 20% Glycerol, 500mM NaCl, 1.5 mM MgCl_2_, 0.2mM EDTA, 0.1% Triton X-100, DTT, PMSF, Protease Inhibitors Cocktail). The nuclei were lysed by intermittent tapping during an incubation of 60 minutes at 4°C. This was then centrifuged at high speed and nuclear proteins were obtained in supernatant. The nuclear and cytosolic protein extract thus obtained was utilized for western blotting.

### Gel diffusion assay

Total cell protein (100 µg) was loaded into wells punched in a 1.5% agarose prepared in digestion buffer (50 mM Tris-Cl (pH 7.4), 150 mM NaCl, 5mM CaCl_2_, 0.02% Brij-45) containing 1 mg/ml gelatin and incubated overnight at 37°C. Zones of gelatin digestion were detected by staining agarose gel in a - solution containing 0.25% Coomassie Brilliant Blue R-250. A standard curve of the enzymatic activity as a function of diameter of digested zone was prepared using trypsin. The gelatinase activity was calculated using the standard plot.

### Gelatin zymography

Total protein samples (40 µg) were mixed with an equal volume of 2X sample buffer (0.005% Bromophenol Blue, 20% glycerol, 4% SDS, 100mM Tris-Cl (pH 6.8)) and subjected to electrophoresis in 10% polyacrylamide gels containing gelatin (1 mg/ml) under non-reducing conditions. Gels were washed with 2.5% Triton X-100 and incubated in digestion buffer (as above) at 37°C for 24 hr. The gels were stained and destained subsequently.

### *in situ* Zymography

Cells were cultured on cover slip and treated under different experimental conditions. After methanol fixation, they were embedded in a mixture of 0.5% agarose and 0.1% fluorescein conjugated gelatin spread on a glass slide and incubated at 37°C for 1 hr in developing buffer (50 mM Tris-Cl (pH 7.4), 150 mM NaCl, 5 mM CaCl_2_, 0.02% Brij-45). The liberation of fluorescent signal as a result of gelatinolytic activity was examined using a fluorescent microscope (Olympus Corporation, Japan). Images were captured at 20X magnification

### Western blotting

Equal quantity of protein (20 µg) from various experimental groups were separated on 10% SDS-polyacrylamide gel and transferred to a polyvinylidenedifluoride (PVDF) membrane. The membrane was blocked in 5% Bovine Serum Albumin (BSA) followed by overnight incubation at 4°C with primary antibody against MMP-9, NF- κB, Lamin-A/C & β-Actin and then with secondary antibody for 1.5 hr at 37°C. The membrane was developed by Enhanced Chemiluminescence (ECL) as described by the manufacturer (Amersham GE Healthcare). The intensity of protein bands was analyzed using NIH ImageJ software. Fold change in expression under different experimental conditions was calculated with respect to control after normalizing the data with β-Actin or Lamin A/C.

### Immunocytochemistry

Cells cultured on coverslips under different experimental conditions were methanol fixed and then blocked for 1 hr at room temperature using 3% BSA followed by incubation with primary antibody (Collagen-IV; MMP-9; NF-κB) for 1 hr at 37°C. They were then incubated in FITC conjugated secondary antibody for 1 hr at 37°C. Nuclei were stained with 4',6-diamidino-2-phenylindole (DAPI) and viewed under fluorescent microscope. Overlay images of DAPI and FITC were created for interpretation of results.

### Statistical analysis

Experiments were carried out in triplicates and repeated three times. All data were expressed as Mean+ SEM and significance was evaluated by student’s T-test as well as two way ANOVA. P value was calculated on comparing the data from control vs. NE-treated group and NE-treated group vs. NE+curcumin-treated group. A value of P<0.05 was considered statistically as significant.

### Source of chemicals

All antibodies were purchased from Santacruz Biotechnology Inc., USA. All chemicals were purchased from Sigma-Aldrich, USA unless or otherwise stated.

## Results

### Curcumin prevents Norepinephrine induced cardiac stress in H9c2 cells

**Figure 1:**
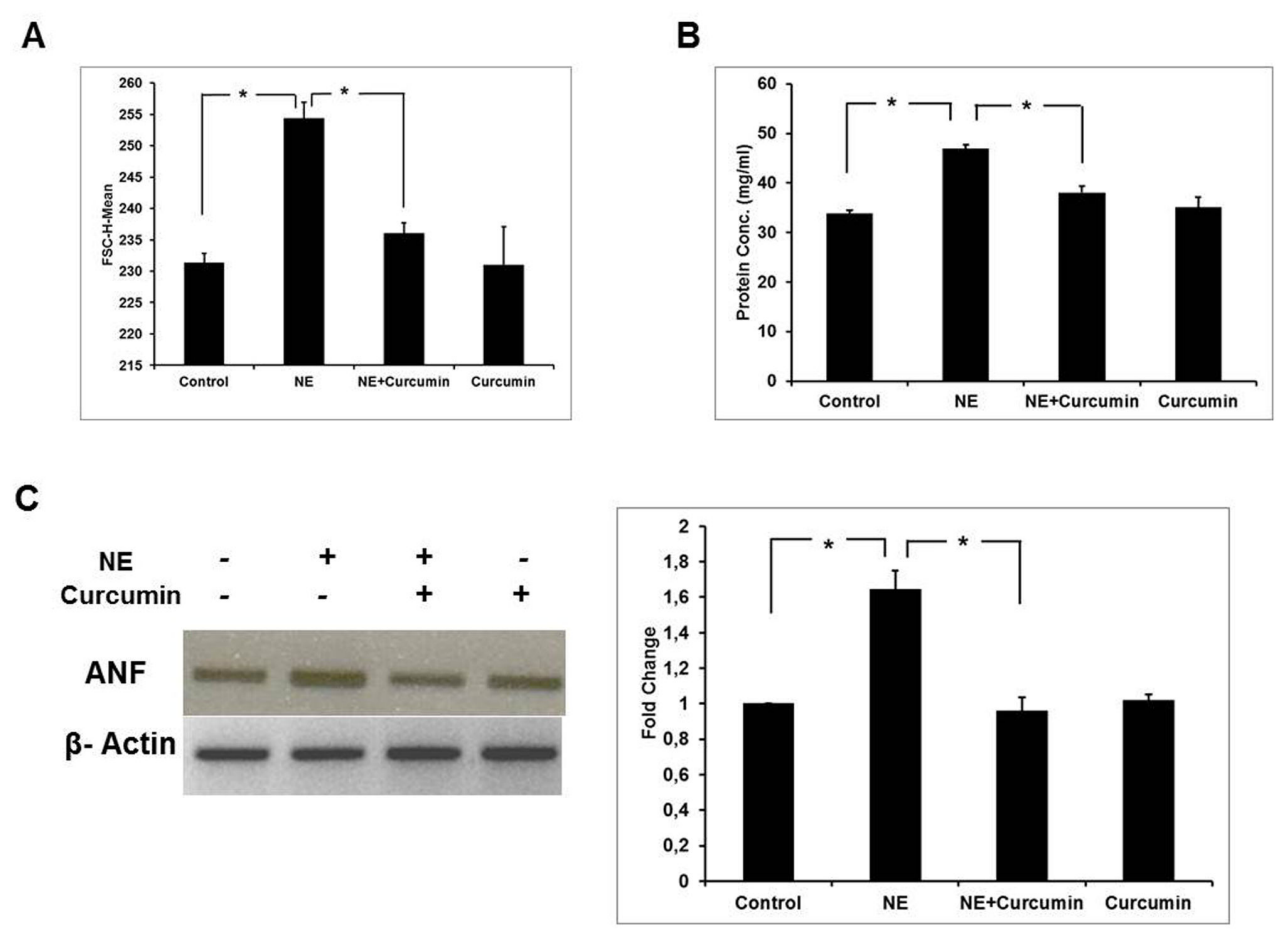
Curcumin prevents Norepinephrine induced cardiac stress in H9c2 cells. **A**) **Analysis** **of** **FACS** **Forward** **Scatter**: Histogram represents comparison of cell size in Control (Uninduced cells), NE-treated (Hypertrophic), NE+Curcumin-treated and CurcuminNE-treated alone experimental groups (*P<0.01). **B**) **Analysis** **of** **protein** **content**: The statistical representation of total protein concentration (mg/ml) for all four groups (*P<0.05). **C**) **RT-PCR** **analysis** **for** **ANF**: Starting from left, samples in different lanes represent, Lane-1: Control; Lane-2: NE-treated; Lane-3: NE+Curcumin-treated; Lane-4: Curcumin-treated. The bands were quantitated by NIH ImageJ software and fold intensity with respect to control after normalization was plotted as a histogram (*P<0.05). The difference in NE-treated group was statistically significant in comparison to control and NE+Curcumin-treated groups in all experiments shown in the figure.

Optimal concentration of curcumin was determined through MTT assay (Figure S1). H9c2 cells were treated with 8 µM curcumin in the presence of 2 µM NE and incubated for 48 hr without any cytotoxicity. Besides this, cells were separately treated with curcumin alone without NE. Increase in size of terminally differentiated cardiomyocytes, protein content and induction of fetal genes such as ANF are indicators of hypertrophic stress [19]. A reduction in the size of cardiomyocytes was observed after treatment with curcumin under hypertrophic conditions as reported earlier by our group (Figure S2). FACS data shows a decrease in the forward scatter (FSC) with curcumin treatment in presence of NE-induced stress indicating a reduction in cell size after curcumin treatment (Figure 1A). A reduction in protein content was also observed on treatment with curcumin in the presence of NE as compared to the cardiomyocytes which were treated with NE alone (Figure 1B). Curcumin treatment along with NE resulted in downregulation of ANF gene expression in comparison to the NE-treated experimental group (Figure 1C). These data suggest that curcumin prevents NE-induced hypertrophic stress in H9c2 cardiomyocytes. Curcumin alone however did not show any significant effect.

### Increase in collagen content due to hypertrophic stress and effect of curcumin

**Figure 2:**
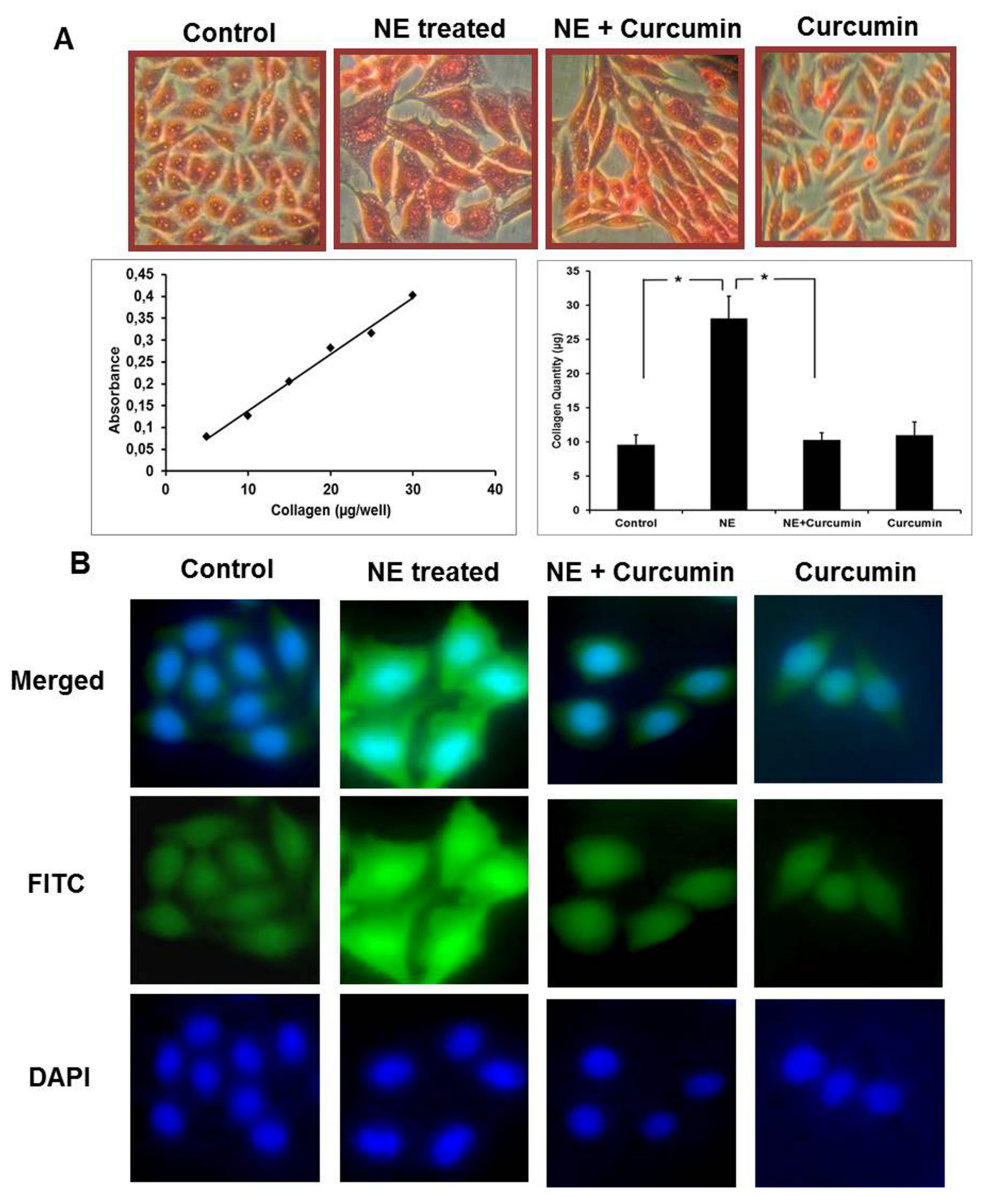
Effect of curcumin on collagen content and expression. **A**) **Analysis** **of** **collagen** **content**: Images captured after Pictrosirus Red staining for (1) Control (2) NE-treated (3) NE+Curcumin-treated (4) Cucumin alone. A standard curve of increasing collagen concentration was plotted as a function of absorbance obtained for the eluted stain. Collagen concentration calculated from standard curve was plotted as a histogram (*P<0.05). NE-treated group showed a significant difference compared to control and NE+Curcumin-treated group. **B**) **Immunocytochemistry** **for** **collagen**: (1) Control, (2) NE-treated (3) NE+Curcumin-treated cells and (4) Cucumin alone.

The total collagen content and the effect of curcumin on it were studied since collagen is a major ECM protein involved in hypertrophic remodeling (Figure 2A). Picrosirius staining analysis showed a 3.5-4 fold increase in collagen content under hypertrophic conditions. Treatment of cells with curcumin in presence of NE, significantly prevented the increase in collagen content which was seen in NE-treated experimental group. Treatment with curcumin alone was however seen to be comparable to control group. Immunocytochemistry was performed to study the expression of collagen-IV, the major collagen involved in hypertrophy (Figure 2B). Similar correlation was observed and curcumin treatment significantly suppressed the expression of collagen-IV which was enhanced under hypertrophic conditions. This emphasizes that the increase in collagen turnover caused due to NE can be prevented by curcumin treatment.

### Inhibition of gelatinolytic activities by curcumin

**Figure 3:**
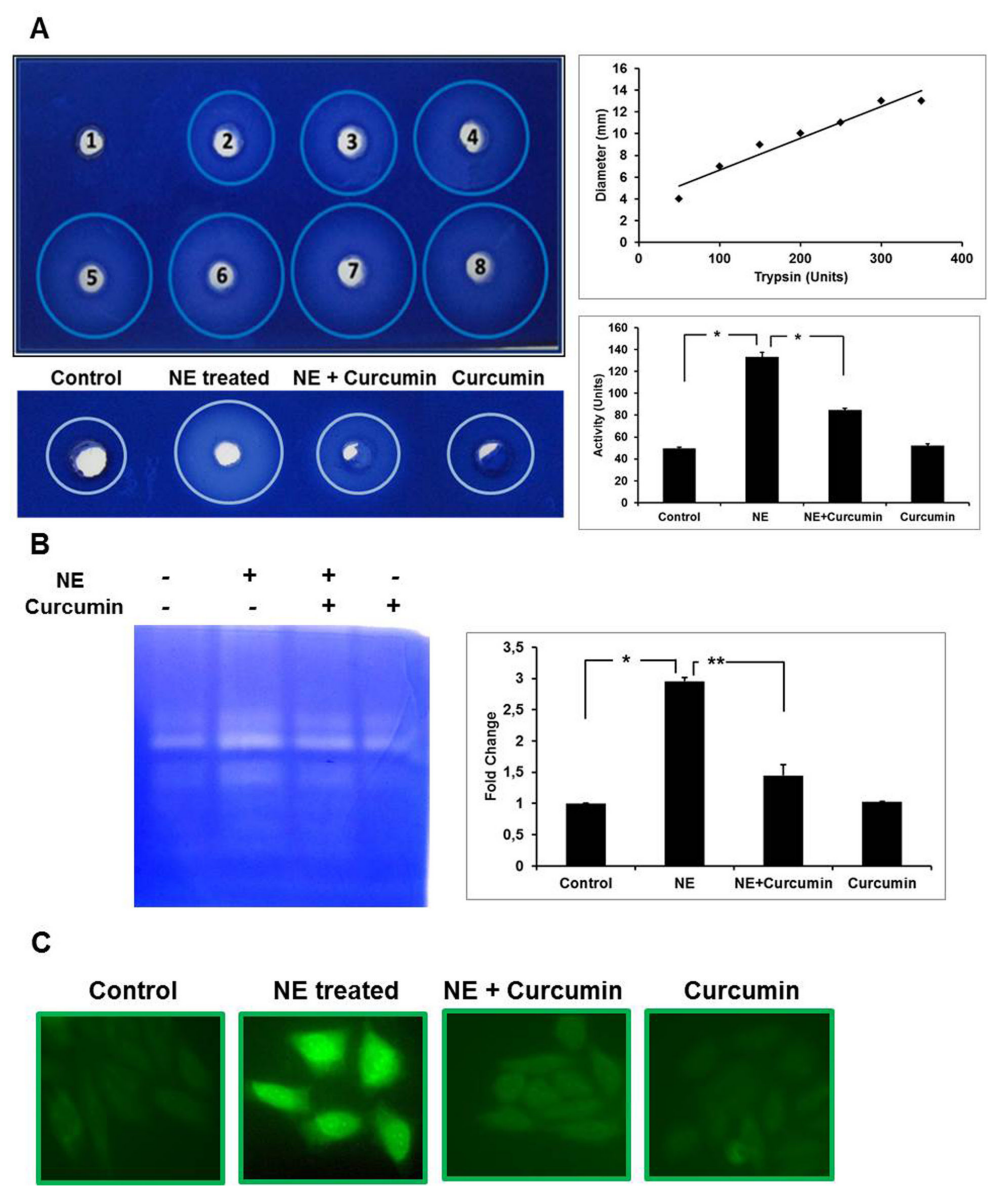
Effect of curumin on gelatinolytic activity. **A**) **Gel-diffusion** **assay**: Upper gel: Various concentrations of trypsin (1-Blank; 2-5 µg/µl; 3-10 µg/µl; 4-15 µg/µl; 5-20 µg/µl; 6-25 µg/µl; 7-30 µg/µl; 8-35 µg/µl) were added to different wells and protease activity was observed as digested zones around it. A standard curve of the enzyme activity in units as a function of diameter of zone was prepared. The enzyme activity for different samples shown in the lower gel (Control; NE-treated; NE+Curcumin-treated; Curcumin-treated alone) was calculated from the standard graph and represented as a histogram (*P<0.01). The difference of NE-treated was significant to control as well as NE+Curcumin-treated group. **B**) **Gelatin** **Zymography**: Samples in different lanes of zymograms starting from the left represented as Lane-1: Control; Lane-2: NE-treated; Lane-3: NE+Curcumin-treated; Lane-4: Curcumin-treated alone. The fold change in the activity for bands corresponding to MMP-9 with respect to control was quantified using ImageJ and plotted as histogram (*P<0.01, **P<0.05). **C**) ***in-situ*** **Gelatin** **Zymography**: The experiment was carried out under different experimental conditions above and images captured by fluoresencent microscope at 20X magnifications are represented.

To compare the activity of gelatinases, a gel diffusion assay was performed using gelatin as substrate. The diameter of the clear zone obtained on digestion provided a direct correlation with the activity of gelatinase. The NE-treated group showed a 2 fold greater clear zone (7 mm diameter) in comparison to cells treated with curcumin in the presence of NE (4 mm diameter), indicating a suppression in the gelatinolytic activity in presence of curcumin (Figure 3A & 3B). Correlation of the activity of 100 µg protein of each group with the standard curve signifies that cardiomyocytes treated with curcumin in presence of NE show an activity of about 85 units of trypsin in comparison to 140 units of trypsin activity observed in NE-treated cells. Cells treated with curcumin alone had an activity of about 52 units of trypsin which was not significantly different from the control group showing an activity of about 49 units.

To further ascertain the effect of curcumin on the activity of gelatinases, gelatin zymography was also carried out with protein extracts. The changes in the activity of gelatinases were found well correlated with the gel diffusion assay. The activity of gelatinase B was enhanced to about three fold under hypertrophic conditions in comparison to control while curcumin treatment along with NE showed an inhibitory effect with only about 1.3 fold increase in the gelatinase B activity compared to control (Figure 3C). The activity shown in the experimental group treated with curcumin alone was comparable to controls. Cell *in-situ* zymography was performed to assess the on-site/in-position functional gelatinolytic activity within the cardiomyocytes. Significant difference was observed in the activity shown by cells treated with NE alone. The activity was inhibited remarkably after curcumin treatment in the presence of NE (Figure 3D). Hence, these results emphasize that curcumin has an inhibitory effect on the activity of gelatinases which was upregulated under NE-induced stress condition.

### Curcumin decreases the expression of Gelatinase B

**Figure 4:**
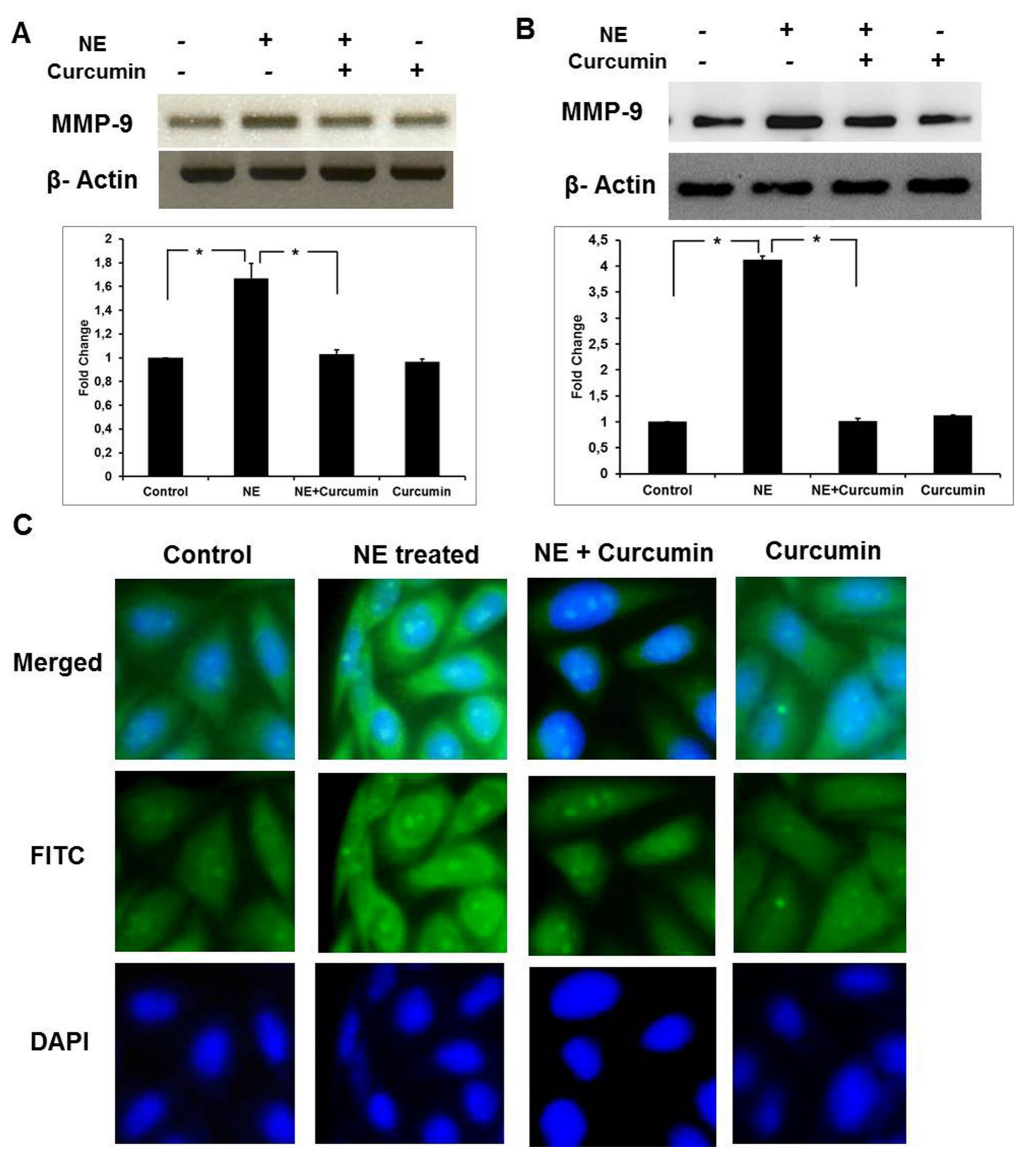
Expression levels of MMP-9 on curcumin treatment. **A**) **RT-PCR** **for** **MMP-9**: mRNA expression seen through semiqunatitative RT-PCR Samples in different lanes starting from the left represented as Lane-1: Control; Lane-2: NE-treated; Lane-3: NE+Curcumin-treated; Lane-4: Curcumin-treated alone. qPCR results obtained were normalized against beta actin and plotted as a histogram (*P<0.05). **B**) **Western** **Blotting** **for** **MMP-9**: Samples in different lanes starting from the left represented as Lane-1: Control; Lane-2: NE-treated; Lane-3: NE+Curcumin-treated; Lane-4: Curcumin-treated alone. Protein expression observed through Western blotting was quantitated by NIH ImageJ software. Results obtained were normalized against beta actin and plotted as a histogram (*P<0.01). **C**) **Immunocytochemistry** **for** **MMP-9**: Images captured by fluoresencent microscope at 20X magnifications are represented. NE-treated group showed a significant difference compared to control and NE+Curcumin-treated group in all experiments shown in the figure.

Of the two members of the gelatinase family, gelatinase B was chosen as the potential candidate for further analysis. To determine the effect of curcumin on the mRNA expression, we performed semi-quantitative RT-PCR analysis. As shown in Figure 4A, curcumin-treated cardiac cells showed significantly lower mRNA expression levels of MMP-9 compared to the NE-induced cells. This was further confirmed by qRT-PCR analysis which showed about negligible increase on treatment with curcumin along NE in comparison to 1.7 fold increase in mRNA expression after NE treatment. To investigate if the downregulation of MMP-9 was also at the protein level, western blot was performed using MMP-9 antibody which showed that curcumin treatment along with NE prevented the increase in the MMP-9 protein expression (Figure 4B). Similar results were also observed in immunocytochemistry experiments (Figure 4C). These results indicate that curcumin suppresses NE-induced MMP-9 expression in cardiomyocytes both at mRNA as well as protein levels.

### Curcumin prevents nuclear localization of NF-κB

**Figure 5:**
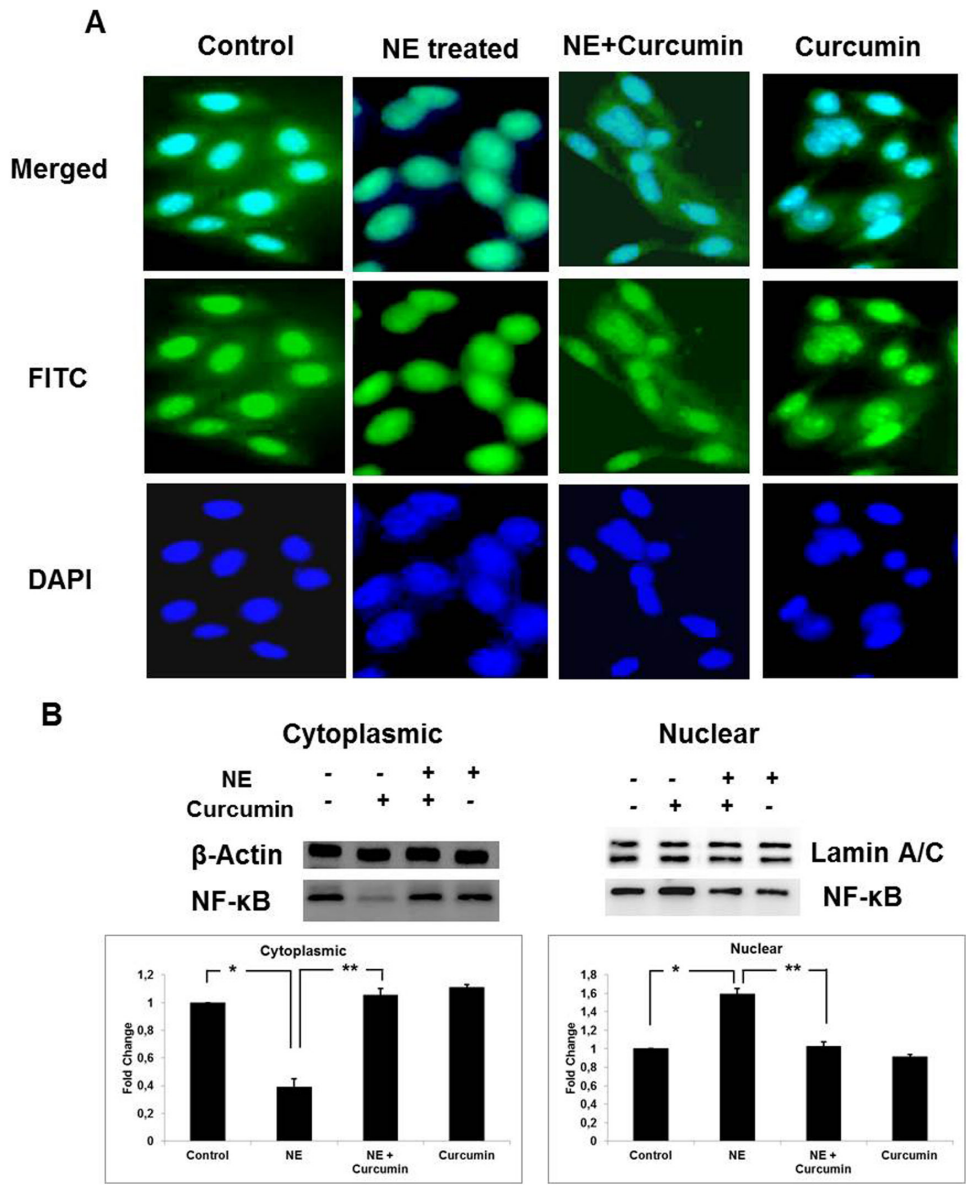
Nuclear localization of NF-κB. (**A**) **Immunofluorescence** **for** **NF-κB**: Nuclear localization of NF-κB was compared between H9c2 Control cells, NE-treated cells NE+Curcumin-treated and Curcumin-treated alone cells. Blue color represents DAPI staining of nucleus. NF- κB was stained green. (**B**) **Western** **Blotting**: Nuclear and cytosolic proteins from different Samples in different lanes for both nuclear and cytosolic extract panels starting from the left represented as Lane-1: Control; Lane-2: NE-treated; Lane-3: NE+Curcumin-treated; Lane-4: Curcumin-treated alone. Protein expression observed through Western blotting was quantitated by NIH ImageJ software. Results obtained were normalized against beta actin for cytoplasmic extracts and Lamin A/C for nuclear extracts and plotted as a histogram (*P<0.01, **P<0.05). NE-treated group showed a significant difference compared to control and NE+Curcumin-treated group.

Gelatinase-B is transcriptionally regulated by NF-κB. In order to find out the effect of curcumin on it, we carried out immunofluorescence studies with NF-κB antibody to determine its localization under different experimental conditions. NE induction resulted in localization of NF-κB inside the nucleus. This possibly triggers the transcriptional machinery and further expression of MMP-9 gene. Curcumin-treated cells showed a reduced localization of NF-κB inside the nucleus. NF-κB was found to be localized majorly in the cytoplasm (Figure 5A). The result was also confirmed by performing western blot of nuclear and cytoplasmic protein extracts separately. Expression of NF-κB was greater in the nuclear extract prepared from NE-induced cells and the cytosolic extracts of cells treated with both curcumin and NE. Control as well as cells treated with curcumin alone also showed cytoplasmic localization (Figure 5B). These findings suggest that curcumin suppresses the entry of NF-κB triggered due to NE, inside the nucleus.

## Discussion

The rationale of the present study is to assess the effect of curcumin on gelatinases under NE-induced stress conditions. In our study, it was observed that curcumin prevented an increase in the collagen content and its expression. Moreover, it could effectively suppress the activity and expression of gelatinase B.

H9c2 cells show almost identical hypertrophic responses to those observed in primary cardiomyocytes and thus can be used as a model for *in vitro* studies of cardiac hypertrophy [20]. Induction of hypertrophic stress is associated with an increase in cell size and expression of marker genes such as ANF. Curcumin has a wide array of pharmacological effects but its role in cardiac hypertrophic remodeling is as yet largely unknown. In our study, H9c2 cells undergoing NE-induced stress show an increase in cell size, protein content and ANF gene expression which is prevented on treatment with curcumin. This apparent difference suggests that curcumin is playing a role in altering the cellular events in H9c2 cells in order to circumvent the hypertrophic stress condition.

Previous studies have shown that cardiac hypertrophy is characterized by an overall imbalance of ECM turnover with myocardial collagen accumulation [21,22]. It has been reported that under cardiac stress, an increase in ventricular collagen deposited leads to a rise in collagen concentration as well as changes in collagen composition [23]. Curcumin treatment of H9c2 cells under hypertrophic conditions prevented the increase in collagen content and expression in cardiomyocytes indicating that curcumin is directly able to target the process of collagen synthesis. The increase in collagen synthesis is associated with an increase in the enzymatic activity of collagen degrading enzymes. Degradation of collagen forms gelatin which is further digested by gelatinases [24]. The enzymatic activity of gelatinases is thus elevated under hypertrophic stress. Henceforth, the effect of curcumin on the activity and expression of gelatin degrading enzymes was evaluated. Activity assays demonstrate that curcumin suppresses the gelatinolytic activity of the proteases from cells undergoing stress. Cell *in situ* zymography, which demonstrates in-position activity of these proteases showed comparable effects to the activity assays.

Collagen turnover and ECM remodeling that occur during various physiological and pathological processes are largely dependent on the regulation of MMP activity [25]. The reduction in ECM remodeling mainly by suppressed MMP activity, further preventing collagen deposition appears to be an attractive therapeutic intervention for heart failure and can be of clinical utility to humans. MMPs, especially gelatinases, are responsible for regulating most of the matrix turnover, since they can collectively degrade the basement membrane proteins like gelatin, collagen type-IV, V and VII, elastin and proteoglycans [26]. MMP activities were known to be up-regulated in cardiac tissues by β-adrenergic stimulation [27]. The gelatin zymography results clearly emphasize that activity of gelatinases is elevated under hypertrophic conditions and is suppressed on treatment with curcumin. An inhibition of ventricular hypertrophy by blocking MMP activity was earlier seen in the TNF-α transgenic mouse model of dilated cardiomyopathy [28]. Use of natural compounds which can inhibit the activity of MMPs has been advocated as a potential therapeutic approach in treatment of cardiovascular disorders [29].

MMP-9 was chosen as a candidate gelatinase for further analysis of the effect of curcumin. MMP-2 is known to be constitutively expressed by many cell types in culture, while MMP-9 expression is induced by cytokines, growth factors, etc. [30]. It is known that MMP-9 activity and expression levels are elevated in cardiomyopathies but still the mechanistic outlook is not very clear [31]. The upregulation of MMP-9 expression in the current study agrees with earlier reported studies [32]. From the present study, it can be suggested that curcumin targets the mRNA as well as protein expression of MMP-9.

The promoter of MMP-9 is highly conserved and carries putative NF-κB binding sites [33]. Hence, there is an involvement of NF-κB in the process of MMP-9 upregulation [34]. By inhibiting NF-κB translocation from cytoplasm to nucleus, the MMP-9 transcriptional pathway is altered which further alters the expression levels of this protein.

**Figure 6:**
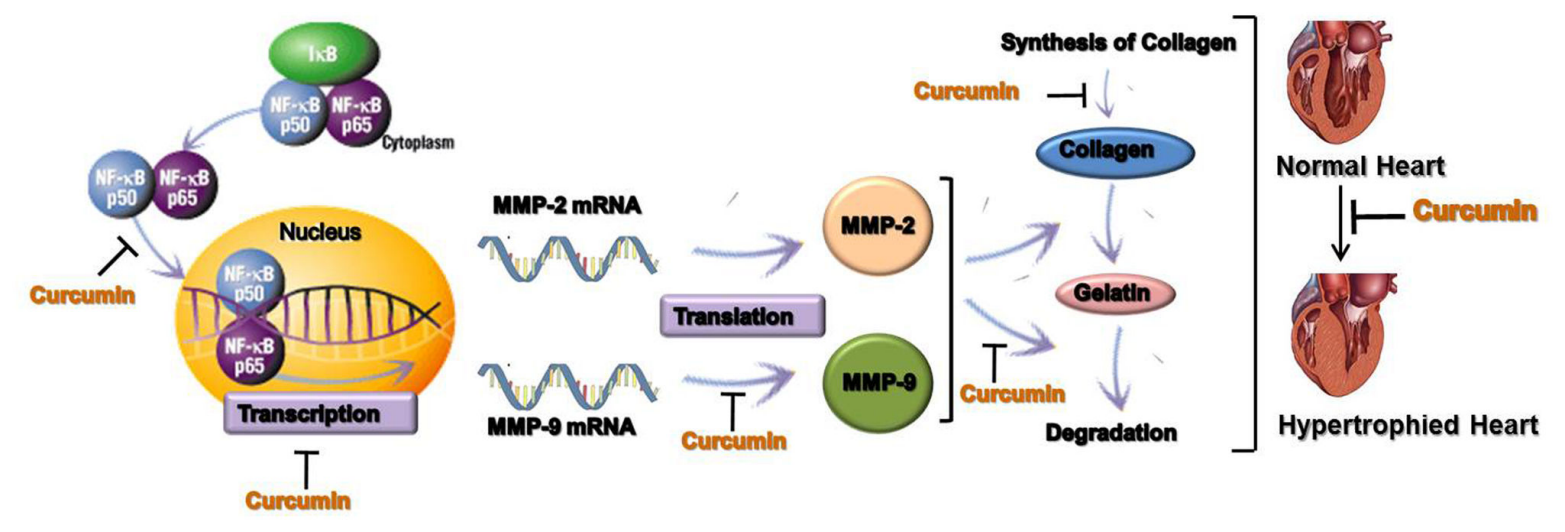
Proposed Model for the study. An illustration of the proposed mechanism of prevention of NE-induced cardiotoxicity by curcumin through downregulation of gelatinase B. It shows that curcumin targets different signaling players in the pathway.

Based on the results of our present work, we propose a mechanism suggesting that the alteration in overall gelatinase pathway due to curcumin could be via targeting the pathway at multiple levels (Figure 6). It could also be hypothesized that curcumin could lead to certain post-translational modifications of gelatinase B which could in turn alter the activity of the enzyme. It is however imperative to know if curcumin is able to directly bind to MMP-9 and result in suppression of its activity. The *in silico* studies performed showed that curcumin has docking sites on the hemopexin domain of MMP-9 (Figure S3). The hemopexin-like domain is known to influence binding of Tissue Inhibitor of Metalloproteinases (TIMPs), certain substrates, and some proteolytic activities [35]. Since curcumin was found to possess docking sites on this domain, it can be hypothesised that curcumin may prevent the substrate binding and suppress the enzymatic activity of MMP-9 by binding to its metal-binding site. However further experimental studies should be performed for its validation. The use of a natural agent which is a MMP inhibitor and cardioprotective can be a potential drug against NE-induced cardiac stress.

A number of MMP inhibitors have been developed over the past few years, however, their design, synthesis, development and testing has been a big challenge and has still given unsatisfactory results. Clinical trials for some synthetic inhibitors such as Ro 32-3555 and BAY 12-9566 (a potent gelatinase B inhibitor) have been suspended [36]. Our study shows that the polyphenol, curcumin, may be established as a potent molecule for suppression of MMP activity. Thus, curcumin can act as a suitable inhibitor that is natural and less toxic in comparison to the chemically synthesized inhibitors, most of which have failed during clinical trials.

In the present work, we have used cultured H9c2 cardiac cells as models to study the NE-induced hypertrophic stress. H9c2 cells provide technical advantages for studying cellular mechanisms, and they have often been used to investigate the pathways implicated in cardiac damage. The extent to which the conclusions of the present work may be applicable to native cardiomyocytes and *in vivo* heart will require further studies using appropriate animal models. This shall confirm the results obtained in the present study.

In conclusion, our study demonstrates that curcumin inhibits NE-induced MMP-9 expression and activity, which in turn suppresses the stress on cardiomyocytes. Our study suggests that curcumin is able to prevent/reverse the stress effects seen in the H9c2 cardiomyocytes due to catecholamines by suppressing gelatinase B.

## Supporting Information

Figure S1 **In vito cytotoxicity of curcumin.** Cells treated with curcumin show about 100% viability till 8 µM urcumin concentration after which it decreases with increasing dose.
(TIF)

Figure S2 **Morphological analysis after curcumin treatment.** Cell size from Control (Uninduced cells), NE treated (Hypertrophic), NE+Curcumin treated and Curcumin treated alone experimental groups was quantified by analyzing images from different fields using ImageJ software and plotted as a histogram (*P<0.01). The difference in NE treated group was statistically significant compared to Control as well as NE+Curcumin treated group.
(TIF)

Figure S3 **Docking of curcumin with gelatinase B.** **A**) **Structure of curcumin. B**) **Molecular docking of curcumin and gelatinase B**: Amino acid residues SER80 and THR577 were found to be critical in docking studies of curcumin and gelatinase B as indicated by the yellow dotted lines.
(TIF)

Supporting Information S1 **Supporting methods.**
(DOC)
