# Supplementary figures and images for: Curcumin Suppresses Gelatinase B Mediated Norepinephrine Induced Stress in H9c2 Cardiomyocytes
Source: PLoS One. 2013 Oct 7;8(10):e76519. doi: 10.1371/journal.pone.0076519 (PMC3792053; doi:10.1371/journal.pone.0076519)

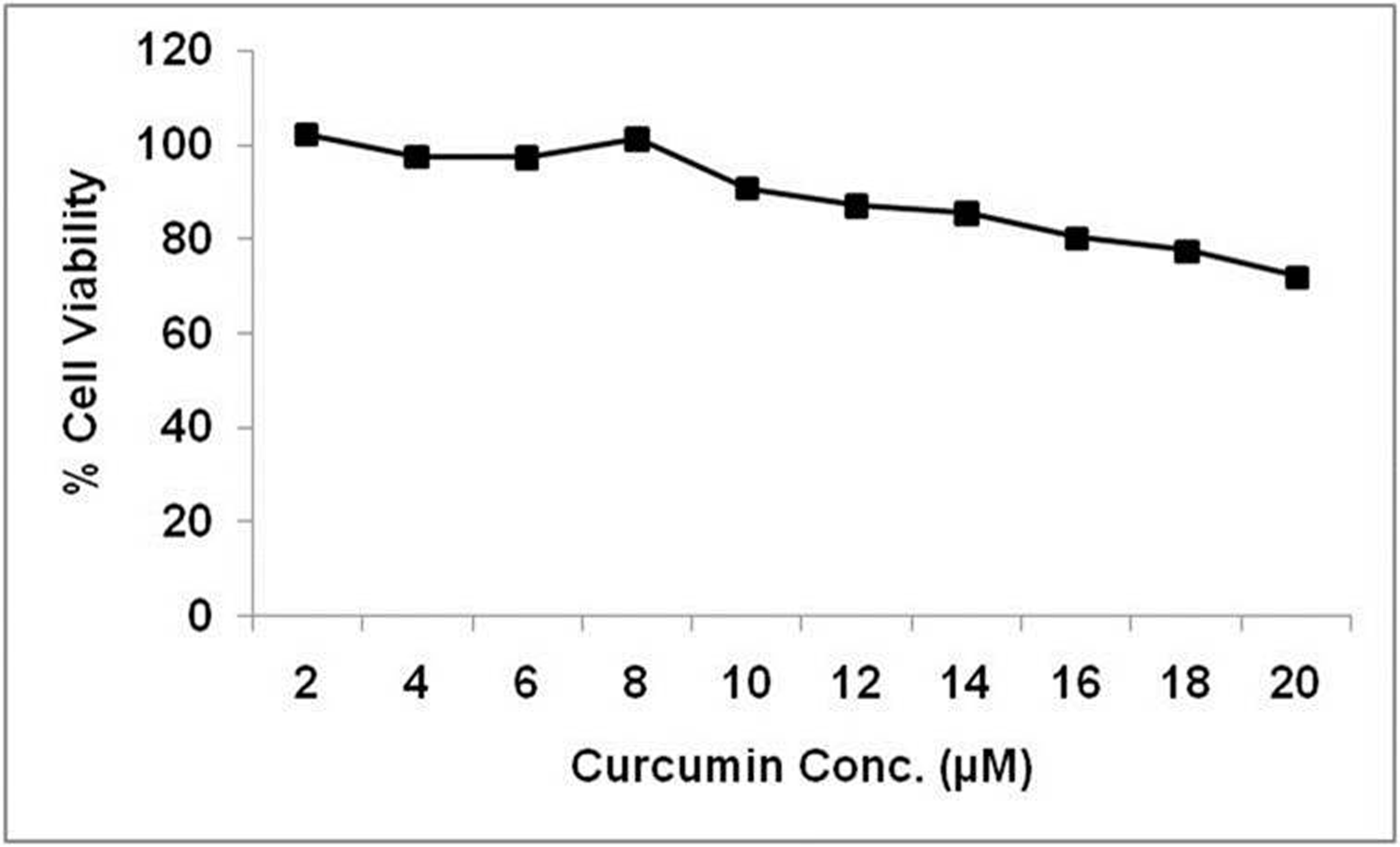

Supplement: Figure S1 — In vito cytotoxicity of curcumin. Cells treated with curcumin show about 100% viability till 8 µM urcumin concentration after which it decreases with increasing dose. (TIF) [file pone.0076519.s001.tif]

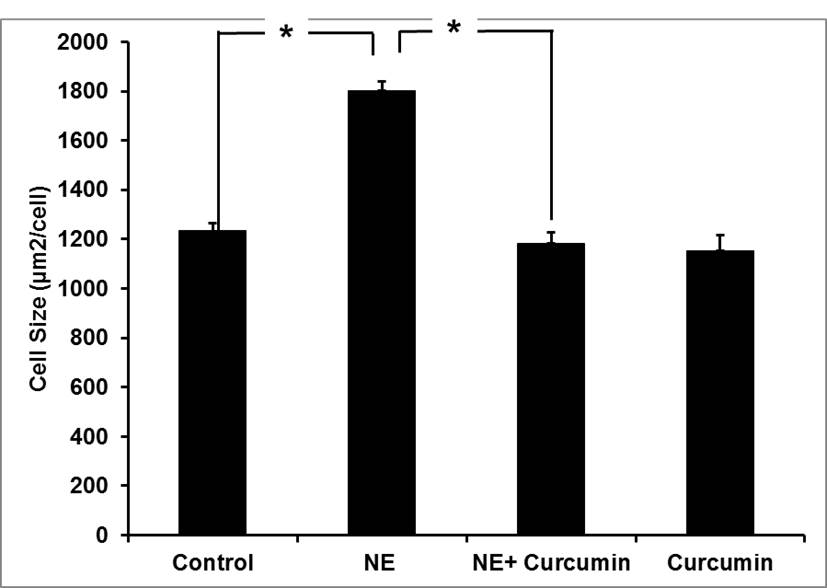

Supplement: Figure S2 — Morphological analysis after curcumin treatment. Cell size from Control (Uninduced cells), NE treated (Hypertrophic), NE+Curcumin treated and Curcumin treated alone experimental groups was quantified by analyzing images from different fields using ImageJ software and plotted as a histogram (*P<0.01). The difference in NE treated group was statistically significant compared to Control as well as NE+Curcumin treated group. (TIF) [file pone.0076519.s002.tif]

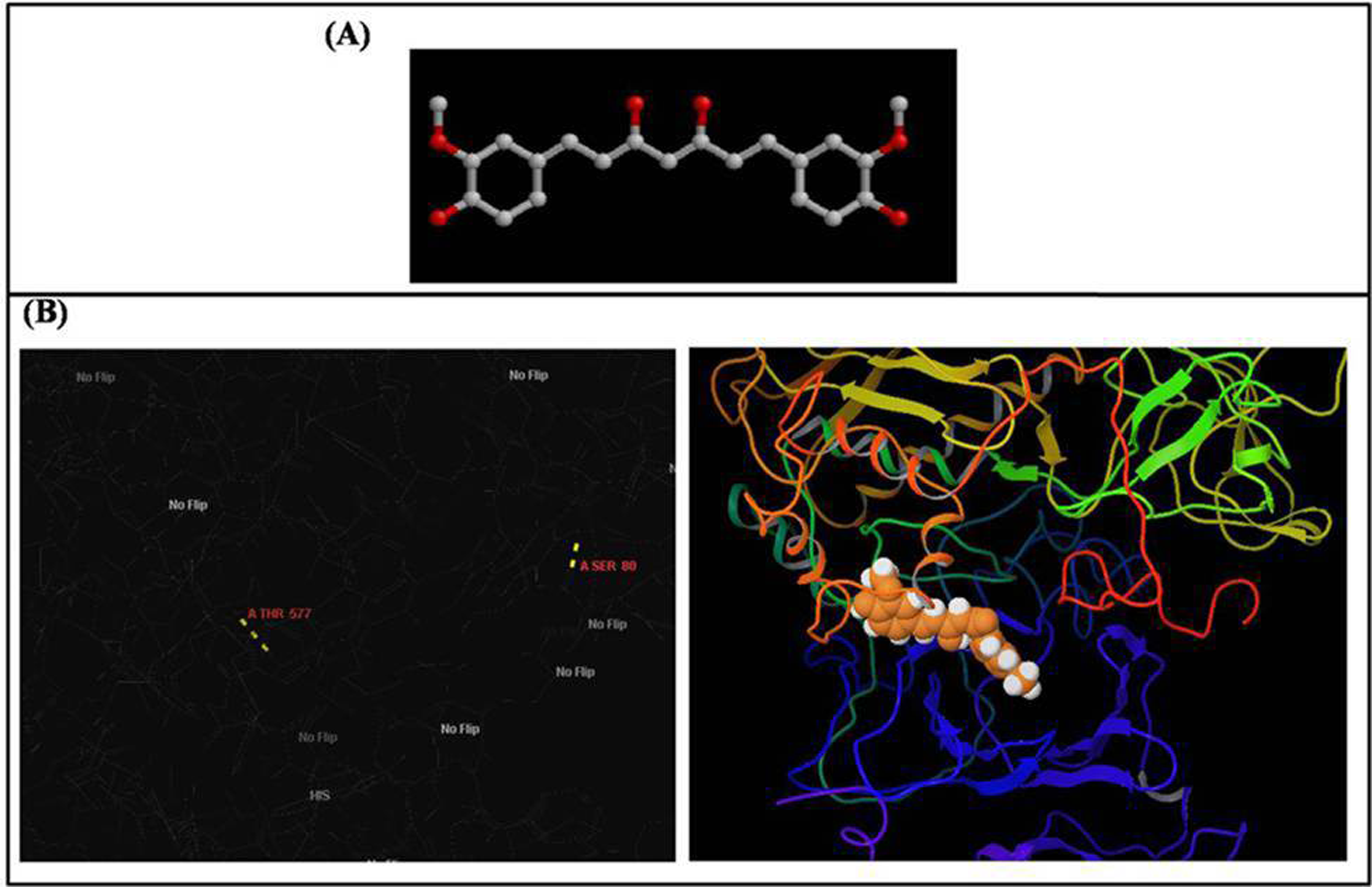

Supplement: Figure S3 — Docking of curcumin with gelatinase B. A) Structure of curcumin. B) Molecular docking of curcumin and gelatinase B: Amino acid residues SER80 and THR577 were found to be critical in docking studies of curcumin and gelatinase B as indicated by the yellow dotted lines. (TIF) [file pone.0076519.s003.tif]
