# Supplementary material for: Curcumin Suppresses Gelatinase B Mediated Norepinephrine Induced Stress in H9c2 Cardiomyocytes
Source: PLoS One. 2013 Oct 7;8(10):e76519. doi: 10.1371/journal.pone.0076519 (PMC3792053; doi:10.1371/journal.pone.0076519)
Supplement: Supporting Information S1 — Supporting methods. (DOC) [file pone.0076519.s004.doc]

***In-vitro* cytotoxicity for curcumin**

*In-vitro* cytotoxicity of curcumin was determined using MTT assay . MTT (3-(4,5-dimethyl-thiazol-2-yl)-2,5-diphenyl tetrazolium bromide) was added after treatment of cells with Curcumin at different concentrations ranging from 2µM to 20µM. Color development was measured after to adding dimethylsulfoxide (DMSO) in ELISA plate reader (Bio-Rad) at 570 nm. Cell viability is defined relative to untreated control cells as follows: cell viability = absorbance of treated sample /absorbance of control.

***In- silico* prediction of molecular targets of curcumin in gelatinase B**

The chemical structure of curcumin was prepared using ChemSketch 12.0 or builder module in Schrödinger. This structure was used for blind docking over the subunits and biologically active domains of gelatinase B enzyme to find the possible interactions for curcumin on the enzyme and correlate its biological functions. Complete sequence structures of gelatinase B downloaded from RCSB protein database (NCBI). These structures were then predicted using I-TASSER server. PDB files were made ligand free, cleaned and optimized using protein preparation wizard tab in Schrödinger. Complete structure outputs of I-TASSER were subjected to optimization and secondary structure prediction using prime tab and docked with curcumin molecule.

In order to predict the binding orientation of curcumin to gelatinase B and thus, predict the affinity and activity of curcumin for a stable interaction with gelatinase B, *in-silico* modeling approach was used to predict the critical residues in Gelatinase B which might be involved in the interaction with curcumin. It was found that the ligand curcumin, docked on SER80 and THR577 on gelatinase B protein. SER80 is present on a peptidoglycan like binding domain while THR577 is present on one of the metal binding site present within the hemopexin domain which suggests that curcumin may exert its effect by blocking the substrate binding of gelatinase B (Fig. S3).
